# Breast self-examination practice and associated factors among pastoralist women in the West Guji Zone, Oromia, Ethiopia: a community-based cross-sectional study

**DOI:** 10.3389/fgwh.2025.1501001

**Published:** 2025-03-20

**Authors:** Mohammed Aliyi, Yimar Hotessa, Abdisa Haro, Belda Negesa Beyene, Misgana Desalegn, Derese Eshetu Debela

**Affiliations:** ^1^ Midwifery Department, College of Medicine and Health Sciences, Madda Walabu University, Shashamane, Ethiopia; ^2^ Department of Midwifery, Institute of Health, Bule Hora University, Bule Hora, Ethiopia; ^3^ Department of Midwifery, College of Medicine and Health Science, Madda Walabu University, Robe, Ethiopia

**Keywords:** breast cancer, breast self-examination, practice, pastoralist women, Guji Zone, Ethiopia

## Abstract

**Background:**

Breast cancer is the most common cancer among women. It is the leading or second cause of female cancer-related deaths in both developed and developing countries, including Ethiopia. Breast self-examination is an effective and efficient screening method used by women for the early detection of breast cancer. There is limited data about breast self-examination practice among pastoralist women in the study area. Therefore, the aim of this study was to assess the magnitude of breast self-examination practice and associated factors among women of childbearing age in the West Guji Zone, South Ethiopia.

**Methods:**

A community-based cross-sectional study was conducted from 1 March to 30 April 2023 on 424 randomly selected women of childbearing age in the West Guji Zone. A systematic sampling technique was employed to select the study participants. Data was collected using pre-tested and structured questionnaires through face-to-face interviews, entered into EpiData version 4.6 and then exported to SPSS version 25 for cleaning and analysis. Bivariable and multivariable analyses were conducted using binary logistic regression to identify factors associated with breast self-examination practice. Statistical significance was declared at a *P*-value <0.05.

**Result:**

In this study, 62 (14.6%) of the women had a good practice of breast self-examination. Maternal age (25–34 years) [adjusted odds ratio (AOR) = 1.98, 95% confidence interval (CI): 1.07–3.70], monthly income (AOR = 3.92, 95% CI: 1.34–11.49), residence (AOR = 2.28, 95% CI: 1.09–4.78), and knowledge about breast self-examination (AOR = 2.15, 95% CI: 1.14–4.05) were factors significantly associated with breast self-examination practice**.**

**Conclusion:**

The study's findings indicated a significantly low level of breast self-examination practice among pastoralist women. Women's education should be promoted, income generated, and the practice of breast self-examination should be advocated.

## 1 Background

Breast cancer is a malignant tumor originating from the breast cells that can range in severity from non-invasive to metastatic carcinoma (1). Breast cancer is a major public health problem, the most common form of cancer in women, and the second leading cause of cancer-related morbidity and deaths next to lung cancer (2). Among women of reproductive age, twice as many breast cancer cases are being diagnosed in low and middle-income countries than in industrialized countries (3). Globally, 1.15 million women are diagnosed with breast cancer, and more than half a million die from this disease every year (4).

There were an estimated 2.1 million newly diagnosed female breast cancer (FBC) cases worldwide in 2018, accounting for one in four cancer cases in women (5). The incidence rates vary greatly worldwide, from 19.3 per 100,000 in Eastern Africa to 89.7 per 100,000 in Western Europe (6). In sub-Saharan Africa, the incidence of breast cancer among women aged 15 and above was 23.5 per 100,000 women (7). In Ethiopia, 24.4% of women have had breast cancer (8). Any delay in the screening of breast cancer and treatment can lead to the diagnosis of the disease at a more advanced stage, leading to an increase in the death rate and a reduction in the chance of survival (9). The low survival rate in developing countries is due to the lack of appropriate early screening programs (10).

The early detection of breast cancer is crucial and involves mammography, a clinical breast examination (CBE), and a breast self-examination (BSE) (11). Of these strategies, BSE is a cost-effective method to detect breast cancer in resource-constrained countries (12). Breast self-examination is a simple, non-invasive, convenient, private, and non-harmful screening method used for the early detection of breast cancer. More than 90% of cases of breast cancer can be detected by women themselves; for this reason, it is important that women should understand the importance of BSE as the key breast cancer detection mechanism (13).

It is recommended that every woman starts BSEs at the age of 20, and it should be carried out once a month for 20 min between the 7th and 10th day of the menstrual cycle to detect breast cancer at the early stages (14).The method involves the woman herself looking at and feeling each breast for a possible mass, discharge, swelling of the breast, skin irritation or dimpling, and any change in size and shape of the breast (15). Regular breast self-examination practice is crucial for the early detection of breast cancer, potentially saving lives and a woman's regular and accurate BSE practice increases her motivation to seek medical attention (4).

Studies have revealed that breast self-examination practice remains low in many African countries. In Nigeria, the prevalence is 12.8% (12), 16.9% in Uganda (16), 16% in Cameroon (17), 8% in Sudan (18), and 6.9% in Ethiopia (19). Knowledge of BSE and breast cancer, income, level of education, ever heard about BSE, fear of detecting cancer, feeling that it violates privacy, and feeling of embarrassment are factors contributing to the low level of breast self-examination practice (7).

The Ethiopian government devotes a great deal of attention and resources to addressing non-communicable, infectious, and communicable diseases. Focus was placed on these illnesses by the government, non-governmental organizations, and international partners. Of these, breast cancer is at the bottom of the priority list (20). Studies have shown that the primary reason women do not undertake breast self-examinations is because they are unaware of the proper technique, timing, and position and because of their attitude toward the practice (19).

Breast cancer prevention is crucial, but early detection methods such as mammography and clinical breast examinations are inaccessible in low- and middle-income countries such as Ethiopia. Furthermore, mammography and clinical breast examinations are expensive as they need trained professionals and regular health facility visits, which are difficult for women living in rural, remote areas of the country (21). BSE is the only feasible approach for wide population coverage as it is a cheap and easy method (10).

Our research considers women of childbearing age because breast cancer is most commonly found in this age group. At this time, the breast cancers are more aggressive and grow faster, and it is the crucial period to detect and prevent breast cancer through breast self-examination screening in this age group. A previous study conducted in the country primarily focused on the health facility level. However, studies done at the community level, particularly in the pastoralist area of the country, are limited, and many women missed early detection and treatment opportunities. Moreover, no previous study has been conducted in the study area. Therefore, the purpose of this study was to assess the level of breast self-examination practice and associated factors among women of childbearing age in the West Guji Zone, Oromia, Ethiopia.

## 2 Methods and materials

### 2.1 Study area, period, and design

The study was conducted in the West Guji Zone, Oromia Region, Ethiopia, from 1 March 1 to 30 April 2023. The area is located 462 km from Addis Ababa, the capital city of Ethiopia, and 300 km from the Ethiopia–Kenya border. The zone has nine woredas and one town administration and a projected population of 1,422,767, of whom 697,156 are male and 725,611 are female. According to the 2007 National Census, there were 265,061 reproductive-age women and 49,370 pregnant women. The zone has 166 health posts, 42 health centers, 2 primary hospitals, and 1 general hospital. A community-based cross-sectional study design was employed.

### 2.2 Populations

All women of childbearing age who lived in the West Guji Zone at least 6 months prior to the study period were considered the source population, while all women of childbearing age who lived in seven randomly selected kebeles in the West Guji Zone during the study period were considered the study population.

### 2.3 Eligibility criteria

Women of childbearing age who lived in the West Guji Zone for the 6 months prior to the study were included in the study, while women who could not provide responses, such as critically ill mothers and women with mental health problems, and female health workers who had knowledge of breast cancer were excluded.

### 2.4 Sample size and sampling procedure

The sample size needed for this study was determined using a single population proportion formula by considering a confidence level of 95%, a margin of error of 5%, a design effect of 1.5%, and a level of breast self-examination practice of 21.3% from the previous study (22), and adding a non-response rate of 10%. The final sample size was 424. The study participants were selected using multistage sampling. First, three woredas were selected from a total of 10 woredas found in the West Guji Zone using simple random sampling by lottery method. In the next stage, kebeles from each selected woreda were randomly selected.

A proportional allocation was then employed to decide the number of households that would be selected from each kebele. To prepare a sampling frame, a list of households in each kebele was used. Finally, households were selected using a systematic random sampling technique. The starting household was selected by the lottery method, and then every Kth household was obtained from their respective sampling frame, and an adequate number of eligible women was obtained based on the sample size proportionally allocated to each kebele.

### 2.5 Data collection tool and techniques

The interviewer-based structured questionnaire was adapted and modified from different peer-reviewed studies in the literature and scientific facts (4, 10, 20, 22). The English version of the questionnaire was translated to Afan Oromo and then back to English to ensure relevance and accuracy. The questionnaires comprise sociodemographic characteristics, history of breast cancer, knowledge of BSE, perception toward BSE, and BSE practice. Seven health extension workers and three supervisors were recruited for data collection. One day of training was given to the data collectors and supervisors. The pre-test was conducted and the data was collected through a face-to-face interview at the participants’ homes.

### 2.6 Study variables

#### 2.6.1 Dependent variable

Breast self-examination practice.

#### 2.6.2 Independent variables

Sociodemographic characteristics including age, occupation, educational status, employment status, income, marital status, religion, residence, marital status, religion, family history of breast cancer, personal history of breast cancer, knowledge of BSE and early danger signs of breast cancer, and risk perception about breast cancer.

### 2.7 Operational definitions and measurements

Good breast self-examination practice was defined as those who performed a breast self-examination a week after each menses using their palm and three middle fingers, otherwise defined as poor practice. Regular BSE practice was defined as performing BSE correctly at least once in a month after menses consistently.

Good knowledge was defined as a mean score or above for the provided 14 closed-ended questions about BSE. Poor knowledge was defined as a score below the mean for the provided 14 closed-ended questions about BSE (22).

A favorable attitude was defined as a score equal to and greater than the mean for the attitude questions. An unfavorable attitude was defined as a score less than the mean for the attitude questions (22). Regarding perceived benefits, scores to the answers to the four questions on the benefit of performing a BSE for the early detection of breast cancer below two were defined as low perceived benefits and above 2 was defined as good perceived benefits (22).

### 2.8 Data quality control

The questionnaires were prepared in English, translated to Afan Oromo, and translated back to English to ensure their originality. A pre-test was conducted on 5% of the sample population at Gerba Kebele, and based on the result, some modifications were made to the questionnaire after the pre-test. Data collectors and supervisors were trained for 1 day to make them familiar with the objective of the study. Data were collected under the supervision of the principal investigator and supervisor. Questionnaires were checked for completeness on a daily basis by supervisors.

### 2.9 Data processing and analysis

The data was entered into EpiData version 4.6 and exported to SPSS version 25 for coding and analysis. Descriptive statistics, including frequencies, percentages, means, and standard deviations, were calculated to describe the characteristics of the study respondents. Bivariate and multivariable analyses were conducted in a binary logistic regression model to identify factors associated with BSE. The assumption of binary logistic regression was checked. The Hosmer and Lemeshow test was conducted to check the final model's fitness. Multicollinearity among independent variables was checked by the variance inflation factor (VIF <10). Adjusted odds ratios (AORs) with a 95% confidence interval (CI) were computed to determine the level of significance. Statistical significance was declared at *P*-value <0.05. The results are presented using tables and figures.

## 3 Results

### 3.1 Sociodemographic characteristics

#### 3.1.1 Family and personal history of breast cancer

A total of 424 reproductive-age women were involved in the study. The mean age (±SD) of study respondents was 28 (±6) years. Of the study participants, 210 (49.5%) were between the ages of 25–34 years; 372 (87.7%) were married; 137 (32.3%) had no formal education, and 224 (52.8%) study respondents were housewives (Table 1). Of the study participants, 357 (84.2%) reported that they did not have a family history of breast cancer, and 67 (15.8%) had a family history of breast cancer. Furthermore, 30 (7.1%) respondents had a personal history of breast cancer and 49 (11.6%) knew someone or patients suffering from breast cancer (Table 2).

**Table 1:** Sociodemographic characteristics of the study participants in the West Guji Zone, Oromia, Ethiopia.

Variable	Frequency	Percentage
Age in completed years		
15–24	134	31.6
25–34	210	49.5
35–49	80	18.9
Residence		
Urban	289	68.2
Rural	135	31.8
Education		
No formal education	137	32.3
Primary (grade 1–8)	121	28.5
Secondary (grade 9–12)	95	22.4
Tertiary and above	71	16.7
Occupation		
Housewife	224	52.8
Government employee	93	21.9
Merchant	76	17.9
Private	31	7.3
Marital status		
Married	372	87.7
Other^a^	52	12.3
Husband’s education		
No formal education	106	25
Primary (grades 1–8)	130	30.7
Secondary (grades 9–12)	100	23.6
Tertiary education and above	88	20.8
Distance from health facility		
Less than or equal to 5 km	239	56.4
Greater than 5 km	185	43.6
Monthly income		
Less than 500	61	14.4
500–1,200	116	27.4
1,201–2,500	104	24.5
2,501–3,500	47	11.1
Greater than or equal to 3,501	96	22.6

^a^ Other: single or separated.

**Table 2:** Family and personal history of breast cancer among women of childbearing age who participated in the study in the West Guji Zone, Oromia region, Southern Ethiopia.

Variable	Frequency	Percentage
Do you have a family history of breast cancer?		
Yes	67	15.8
No	357	84.2
If yes, who is affected?		
Mothers	12	2.8
Sister	18	4.2
Grandmothers	20	4.7
Aunt	17	4
Do you have a personal history of breast cancer?		
Yes	30	7.1
No	394	92.9
Do you know someone suffering from breast cancer?		
Yes	49	11.6
No	375	88.4

### 3.2 Knowledge of and attitude toward breast self-examination of the study participants

Of the study respondents, 142 (33.9%) participants had good knowledge of breast self-examination practice. Furthermore, 209 (49.5%) of the total respondents had ever heard about BSE and 324 (76.4%) participants had begun BSE after 20 years of age (Table 3).

**Table 3:** Knowledge of breast self-examination practice of women of childbearing age who participated in the study in the West Guji zone, Southern Ethiopia, in 2023 and associated factors.

Variable	Frequency	Percentage
Do you know that early detection of breast cancer can improve the chance of survival?		
Yes	252	59.4
No	172	40.6
Do you know about the presence of breast cancer screening methods?		
Yes	231	54.5
No	193	45.5
Do you know the different types of breast cancer screening methods?		
Yes	166	39.2
No	258	60.8
Types of screening methods known		
BSE	81	48.8
CBE	75	45.2
Mammography	10	6
Have you ever heard about BSEs?		
Yes	209	49.3
No	215	50.7
Where did you hear about BSEs?		
Television	88	36.7
Radio	18	7.5
Newspaper/magazines	13	5.4
Health professional	95	39.6
Breast cancer patients	26	10.8
Who should perform BSEs?		
Women	407	96
Men	6	1.4
Both	11	2.6
When should women begin BSEs?		
At an age younger than 20 years	100	23.6
At an age of 20 years and above	324	76.4
How often should a BSE be performed?		
Once a week	118	27.8
Once a month	101	23.8
Once in 3 months	124	29.2
Once in 6 months	81	19.1
What do we look for during a BSE?		
Breast lump	125	29.5
Change in the size of the breast	81	19.1
Change in the nipple and unusual discharge	157	37
Change in the skin color of the breast	50	11.8
Other	11	2.6
Where will you go if you find any symptoms of breast cancer?		
Health facility	348	82.1
Traditional healer	60	14.2
Private clinic	14	3.3
Other	2	0.5
What are the advantages of regular BSE practice?		
Detect any abnormality in the breast	174	41.1
To learn how the breast normally looks and feels	108	25.5
To detect breast cancer earlier and promote treatment	142	33.5
Knowledge		
Good	142	33.9
Poor	282	66.5

Of the study respondents, 285 (67.2%) of the study participants had a favorable attitude toward breast self-examination practice and 139 (32.8%) had an unfavorable attitude (Table 4).

**Table 4:** The attitude toward breast self-examination practice of women of childbearing age who participated in the study in the West Guji Zone, Southern Ethiopia, in 2023 and associated factors.

Predictor variable	Strongly agree	Agree	Neutral	Disagree	Strongly disagree
	*N* (%)	*N* (%)	*N* (%)	*N* (%)	*N* (%)
BSEs are a low-cost method for detecting breast cancer early	73 (17.2)	257 (60.6)	74 (17.5)	16 (3.8)	4 (0.9)
I believe it is very useful to examine my breasts regularly	100 (23.6)	269 (63.4	28 (6.6)	27 (6.4)	
Doing a BSE will make me worry about breast cancer	68 (16)	215 (50.7)	57 (13.4)	74 (17.5)	10 (2.4)
Doing a BSE will take too much time	17 (4)	38 (9)	81 (19.1)	250 (59)	38 (9)
I do not have enough privacy to do a BSE	5 (1.2)	50 (11.8)	90 (21.2)	246 (58)	33 (7.8)
I am confident I can perform a BSE regularly	20 (4.7)	150 (35.4)	114 (26.9)	123 (29)	17 (4)
It is not difficult for me to remember to do a BSE regularly	15 (3.5)	174 (41)	120 (28.3)	96 (22.6)	19 (4.5)
I am sure of the steps to follow to do a BSE	9 (2.1)	138 (32.5)	128 (30.2)	138 (32.5)	11 (2.6)
Attitude					
Favorable	*N* = 285	67.2%			
Unfavorable	*N* = 139	32.8%			

### 3.3 Breast self-examination practice of the study participants

Of the study participants, 62 (14.6%) women had good breast self-examination practice. Furthermore, 39 (9.2%) study participants were doing a breast self-examination regularly, and 92 (21.7%) knew the appropriate time to perform a breast self-examination (Table 5).

**Table 5:** Assessment of BSE practice among women of childbearing age in the West Guji Zone, Southern Ethiopia, in 2023.

Variable	Frequency	Percentage
Did you perform a BSE?		
Yes	94	22.2
No	330	77.8
Why did you perform a BSE?		
Had a previous breast problem	17	4
Fear of breast cancer from family history	15	3.5
Recommended by a health professional	157	37
For early detection and treatment	129	30.4
Fear of developing breast cancer	106	25
How often do you practice BSEs?		
Once a week	38	9
Once a month	78	18.4
Once in 3 months	20	4.7
Once in 6 months	38	9
When it comes to my mind	66	15.6
I don't know	184	43.4
How is a BSE done?		
With the palm and three middle fingers	110	25.9
Palpate with any of the fingers	109	25.7
I don't know	205	48.3
When do you perform a BSE?		
2–3 days after menstruation	92	21.7
When it comes to my mind	80	18.9
On regular days each month	39	9.2
Few days before menses	111	26.2
At any time in each month	102	24.1
How many times in the last 12 months did you perform a BSE?		
10–12 times	39	9.2
7–9 times	60	14.2
4–5 times	61	14.4
1–3 times	83	19.6
Did not perform BSE in the last 12 months	181	42.7
Reason for not practicing BSE		
Forgetfulness	25	5.9
Too busy and did not have enough time	3	0.7
Performing it causes discomfort	15	3.5
Fear of finding something or having breast cancer	55	13
Do not know how to do it	294	69.3
Not convinced about its effectiveness	14	3.3

### 3.4 The magnitude of and factors associated with breast self-examination practice

In this study, the magnitude of breast self-examination practice among reproductive-age women was 14.6% (95% CI: 11.4%–18.4%). In the multivariable logistic regression analysis, after controlling for possible confounding variables, the age, residence, income, and knowledge variables were significantly associated with breast self-examination practice at a *P*-value <0.05. According to the findings of this study, breast self-examination was 1.98 times more likely to be practiced among reproductive women aged 25–34 years (AOR = 1.98; 95% CI: 1.06–3.70) than those aged 15–24 years.

In this study, women who had earned an average monthly income of between 2,501 and 3,500 Ethiopian birr (AOR = 3.92; 95% CI: 1.34–11.48) were 3.92 times more likely to practice BSE compared to women who had a monthly income less than 500 Ethiopian birr. Women who were urban residents (AOR = 2.28; 95% CI: 1.09–4.78) were 2.28 times more likely to practice breast self-examinations compared to women who were rural residents. The odds of practicing breast self-examination were 2.15 times higher among women who had good knowledge (AOR = 2.15; 95% CI: 1.14–4.05) of BSE compared to women who had poor knowledge of BSE (Table 6).

**Table 6:** Bivariate and multivariate logistic regression analysis of BSE practice among the study participants in the West Guji Zone, Southern Ethiopia.

Variable	Ever performed a BSE				*P*-value
	Yes	No	COR (95% CI)	AOR (95%CI)	
Age in completed years					
15–24	18 (13.4%)	116 (86.6%)	1	1	
25–34	39 (18.6%)	171 (81.4%)	0.43 (0.15–1.20)	1.98 (1.06–3.70)^a^	0.03
35–49	5 (6.2%)	75 (93.8)	0.29 (0.11–0.77)	0.34 (0.11–0.99)^a^	0.049
Education					
No formal education	11 (8%)	126 (92%)	1	1	
Primary (grades 1–8)	18 (14.9%)	103 (85.1%)	2.33 (0.97–5.58)	1.44 (0.62–3.35)	0.393
Secondary (grades 9–12)	21 (22.1%)	74 (77.9%)	1.16 (0.52–2.58)	1.84 (0.77–4.37)	0.168
Tertiary and above	12 (16.9%)	59 (83.1%)	0.71 (0.32–1.57)	1.09 (0.36–3.29)	0.867
Household average monthly income in birr					
<500	6 (9.8%)	55 (90.2%)	1	1	
501–1,200	11 (9.5%)	105 (90.5%)	0.95 (0.32–2.81)	0.70 (0.23–2.07)	0.521
1,201–2,500	19 (18.3%)	85 (81.7%)	0.98 (0.39, 2.49)	1.52 (0.55–4.18)	0.418
2,501–3,500	17 (36.2%)	30 (63.8%)	0.46 (0.19–1.08)	3.92 (1.34–11.48)^a^	0.013
>3,501	9 (9.4%)	87 (90.6%)	0.18 (0.074, 0.45)	0.51 (0.16–1.64)	0.262
Residence					
Urban	51 (17.6%)	238 (82.4%)	2.41 (1.21–4.8)	2.28 (1.09–4.783)^a^	0.029
Rural	11 (8.1%)	124 (91.9%)	1	1	
Know someone suffering from breast cancer					
Yes	4 (8.2%)	45 (91.8%)	2.05 (0.71–5.94)	0.43 (0.14–1.35)	0.151
No	58 (15.5%)	317 (84.5%)	1	1	
Knowledge about BSE					
Good	28 (19.7%)	114 (80.3%)	0.55 (0.32–0.96)	2.15 (1.14–4.05)^a^	0.018
Poor	34 (12.1%)	248 (87.9%)	1	1	

^a^ A statistically significant variable at *p* < 0.05.

## 4 Discussion

Breast cancer is the most prevalent cancer among women, particularly in developing countries. A breast self-examination is one of the screening methods in which a woman examines her breasts for any abnormal changes. There was limited data on BSE in the study area. Therefore, the aim of this study was to assess the practice of breast self-examination and its associated factors among women of childbearing age in the West Guji Zone, Oromia, Southern Ethiopia, in 2023.

This study revealed that the magnitude of breast self-examination practice among reproductive-age women was 14.6% (62; 95% CI: 11.4–18.4), which was consistent with the study done in Jimma with 15% (10) and the Bale Zone with 13.2% (21). This could be due to the similar socioeconomic characteristics of the study populations. However, the prevalence was lower than in studies conducted in Ghana (37.6%) (23) and Ethiopia (32.6%) (24). However, it was higher than studies conducted in Eritrea (11.7%) (25) and northern Ethiopia (6.5%) (20). This discrepancy could be a result of differences in sample size, study period, populations, economic status, and geography.

In this study, 67.2% of the study respondents had a favorable attitude toward breast self-examination practice. This finding was supported by 4.7% of women who were confident that they could perform a breast self-examination regularly every month. Furthermore, 15.8% of the study participants had a family history of breast cancer. In this study, 4.7% of the participants’ grandmothers had been affected by breast cancer and 7.1% of the cases were related to the personal history of breast cancer of the study participants.

In this study, maternal age was statistically significant with breast self-examination practice. This finding was supported by studies conducted in Bangladesh (26), Cameroon (17), and Ethiopia (10, 21, 27). This implies that as age increases, the prevalence of breast self-examinations also increases. This may be due to the fact that at this age, women are paying attention to their reproductive activity, and, as a result of increased contact with health facilities and health professionals than at any other time in their lives, their attention to and care of their breast health increases.

According to these study findings, average monthly income was statistically associated with breast self-examination practice. This finding was in line with studies conducted in Palestine (28) and Jimma (10). This could be due to the fact that women who have a higher income level can visit health facilities and they might have access to information about the early practice of BSE.

In this study, the residence of the study participants affected the prevalence of breast self-examinations among reproductive-aged women. This finding was consistent with studies conducted in Uganda (16) and Jimma, Ethiopia (10). This may be due to mothers who lived in urban areas having access to information about BSE practice and the accessibility of health facilities and health professionals, and, thus, they are aware more about BSE practice. Breast self-examination practice was significantly affected by the knowledge of the study respondents. This finding was supported by studies conducted in Uganda (16) and Ethiopia (7, 29). This might be due to the awareness levels of the study respondents.

### 4.1 Strengths and limitations of this study

For breast self-examination practice, each variable was managed independently to control the effect of confounders and prevent bias from being introduced at the analysis stage. Furthermore, the results can be generalized for the total population of the study area. The responses of the study participants were based on self-reported information, which is subject to bias since the study raised personal issues; thus, there is the possibility of underestimation or overestimation. In addition, the study only included women of reproductive age, defined as those between the ages of 15 and 49; women over the age of 49 were not considered. The use of a cross-sectional design has limited the degree of cause-and-effect associations that can be made among the variables of interest.

## 5 Conclusion

The practice of breast self-examination among women of childbearing age in the West Guji Zone was low. Factors such as maternal age, income, residence, and knowledge were the factors associated with breast self-examination practice. Health professionals should raise awareness of and provide health education on BSE to reproductive-age mothers. The government should promote income-generating mechanisms for women and ensure the accessibility of information through different media outlets.

## Data Availability

The original contributions presented in the study are included in the article/Supplementary Material, further inquiries can be directed to the corresponding author.
